# Effects of Muscle-Strengthening Intervention Exercise on Pain Alleviation and Postural Control in Patients with Chronic Low Back Pain

**Published:** 2019-01

**Authors:** Sungyung CHUN, Kyoungkyu JEON

**Affiliations:** 1. Department of Football Science, School of Health Science, Honam University, Gwangju, Republic of Korea; 2. Division of Sport Science, College of Arts and Physical Education, Incheon National University, Incheon, Republic of Korea; 3. Sport Science Institute, Incheon National University, Incheon, Republic of Korea

## Dear Editor-in-Chief

The pelvis is structurally symmetrical in the mid-sagittal plane and connects the lumbar spine and lower extremities, it has effects on static and dynamic postural control (1). Moreover, a shift in the center of mass due to lumbar lordosis and increased anterior pelvic tilt leads to a higher risk of falls, due to impaired balance and dynamic movement (2). Thus, to maintain a healthy thoracolumbar and lumbosacral curvature, conservative treatment using various muscle strengthening and resistance exercises can help with postural control by ensuring symmetrical mobility and proper spine alignment. It is important to stabilize the spine by using proactive conservative exercise therapy to strengthen the muscles involved in postural control and correct malalignment of the body (3). Therefore, this study aimed to improve public health for comfortable daily living by investigating the positive effects of resistance exercise for alleviating chronic low back pain and correcting physical malalignment of the spine and pelvis due to lack of muscle strength in the lumbar region.

The subjects comprised 19 adult males (age 26.42±3.72 year, height 176.29±5.24 cm, weight 75.37±4.81 kg) prescribed functional improvement of malalignment correction for chronic thoracolumbar or lumbosacral pain by a specialist at the exercise center of the hospital in 2016. All participants provided written informed consent, and the study was approved by the Institutional Review Boards at Incheon National University.

To examine the effects of trunk muscle strength improvement following the exercise intervention for pain alleviation, we used an isokinetic muscle and joint testing device (Humac Norm Testing & Rehabilitation, CSMi Solutions, USA) to analyze the peak torque at the lumbar joints. We took 5 measurements at 60 °/sec for each movement. We used a three-dimensional spinal structure analyzer (Formetric 4D, DIERS International GmbH, Schlangenbad, Germany) to measure and evaluate physical alignment. This enables scientific analysis of the spinal curvature and pelvic rotation and tilt by measuring the posterior aspect of the body, including the shape of the spine and pelvis, in the anatomical position. With regard to the accuracy of the measurements, meaning that this method can be used in place of radiographs. We used muscle-strengthening resistance exercises and evaluated the changes in spinal structure and pelvis position and the improvements in muscle function. We initiated a conservative resistance exercise for gradual return to activity accounting for spinal muscle strength, coordination, proprioception, and stability. The subjects participated in a routine consisting of warm-up (Fixed bicycle, Static and dynamic stretching, 10 min), the main exercise (Strength Exercise (Free weight machine), and Prop Exercise (Thera and tubing band, Fitter slide, Balance board, 40 min)), and cool-down (Static and dynamic stretching, 10 min) for 60 min, twice a week, for 8 wk. The means and standard deviations of all the measured data were derived using SPSS 23.0 (IBM Corp, Armonk, NY, USA). A Paired samples t-test were used to analyze differences after 8 week of the resistance exercise program, and the statistical significance level was set to α=.05.

Our results showed that following 8 week of muscle-strengthening resistance exercise, in iso-kinetic testing of the muscles and joints of the lumbar spine, the peak muscle strength of extensor and flexor muscles improved significantly, while in the spinal structure analysis, lateral pelvic tilt, kyphosis angle, lordosis angle, and scoliosis angle all showed statistically significant improvements (Table 1).

**Table 1:** Changes of isokinetic strength and spinal structure

***Variables***		***Pre-exercise***	***Post-exercise***
Isokinetic strength (Nm)	Extensors	207.94±52.17	258.16±63.26 ^*^
	Flexors	186.38±37.61	221.65±42.32 ^*^
Spinal Structure (°)	Pelvic Tilt	2.71±1.57	1.43±1.24 ^*^
	Pelvic Inclination	19.63±3.69	18.27±4.03
	Pelvic Torsion	−.62±2.13	−.43±2.51
	Kyphotic Angle	48.26±6.82	36.61±7.83 ^*^
	Lordotic Angle	41.38±7.31	34.75±5.74 ^*^
	Scoliosis Angle	15.73±3.64	10.62±3.27 ^*^

Values are Mean±SD,

* P<0.05,

** P<0.01

These results are consistent with the results of previous studies that strongly recommend exercise, even after surgical treatment, to enhance recovery of stability, function, and muscle strength impaired as a result of degenerative change in the thoracolumbar or lumbosacral spine (4, 5). Therefore, for pain alleviation and functional recovery in patients with chronic low back pain, proactive muscle-strengthening resistance exercise not only improves muscle strength but also has a positive effect on stability of postural control. It can be an important factor in helping rehabilitation therapy for various abnormal lesions of the thoracolumbar and lumbosacral spine.
